# Pre-pregnancy body mass index and gestational weight gain and their effects on pregnancy and birth outcomes: a cohort study in West Sumatra, Indonesia

**DOI:** 10.1186/s12905-017-0455-2

**Published:** 2017-11-09

**Authors:** Hora Soltani, Nur I. Lipoeto, Frankie J. Fair, Karen Kilner, Y. Yusrawati

**Affiliations:** 1 Centre for Health and Social Care Research, Sheffield Hallam University, Mundella House, 34 Collegiate Crescent, Collegiate Campus, Sheffield, S10 2BP England; 2 Faculty of Medicine, Andalas University, Padang, West Sumatra Indonesia; 3 Department of Obstetrics and Gynaecology, Faculty of Medicine, Andalas University, Padang, West Sumatra Indonesia

**Keywords:** Maternal BMI, Gestational weight gain, Pregnancy outcomes, Birthweight, Indonesia, Cohort study

## Abstract

**Background:**

Indonesia has a considerably high incidence of maternal and infant mortality. The country has however been experiencing a social and economic transition, influencing its general population demographics and nutritional status including the state of health and nutrition of pregnant women. This study aimed to explore body mass index (BMI) and gestational weight gain (GWG), and their relationship with pregnancy outcomes in a sample of Indonesian pregnant women.

**Methods:**

This observational cohort study included a total of 607 pregnant women who were recruited in 2010 from maternity clinics in Western Sumatra, Indonesia. Multiple logistic and regression analyses were undertaken to compare pregnancy and birth outcomes for different BMI and GWG, using normal weight women and women with a recommended weight gain as the referent groups.

**Results:**

The prevalence of underweight (BMI < 18.5 kg/m^2^) in pregnancy was high at 20.1%; while 21.7% of women were overweight (BMI: 23.0–27.4 kg/m^2^) and 5.3% obese (BMI ≥ 27.5 kg/m^2^) using the Asian BMI classifications. The incidence of overweight (BMI: 25.0–29.9 kg/m^2^) and obese (BMI ≥ 30.0 kg/m^2^) according to the international BMI classifications were 13.5% and 1.1% respectively.

The majority of women gained inadequate weight in pregnancy compared to the Institute of Medicine (IOM) recommendations, especially those who had a normal BMI. Birthweight adjusted mean difference aMD (95% confidence interval) 205 (46,365) and the odds of macrosomia adjusted odds ratio aOR 13.46 (2.32–77.99) significantly increased in obese women compared to those with a normal BMI. Birthweight aMD -139 (−215, −64) significantly decreased in women with inadequate GWG compared to those with recommended GWG, while SGA aOR 5.44 (1.36, 21.77) and prematurity aOR 3.55 (1.23, 10.21) increased.

**Conclusions:**

Low nutritional status and inadequate GWG remain a cause for concern in these women. The higher odds of macrosomia with increasing maternal BMI and higher odds of prematurity and small for gestational age infants with inadequate weight gain also require attention.

Research and practice recommendations: Urgent attention is required by researchers, policy makers and decision-makers to facilitate development of culturally sensitive interventions to enhance nutritional status and health of mothers and babies, in an area known for its high incidence of maternal and neonatal mortality.

**Electronic supplementary material:**

The online version of this article (10.1186/s12905-017-0455-2) contains supplementary material, which is available to authorized users.

## Background

Maternal pre-pregnancy BMI is known to influence pregnancy and birth outcomes. Women who are underweight pre-pregnancy have been suggested to have a higher risk of preterm delivery, small for gestational age (SGA) and low birthweight (LBW) [1, 2]. On the other hand, women who are overweight or obese have been shown to have higher rates of induction, instrumental delivery [3], caesarean section (CS) [3, 4], large for gestational age (LGA) and macrosomic infants [2], postpartum haemorrhage, postnatal infection [3] and maternal mortality [5]. Neonates of obese women are also less likely to successfully breastfeed [3, 6] and more likely to be admitted to a neonatal special care unit [3]. Underweight, overweight and obese pregnant women in comparison to those with a normal BMI have a higher number of admissions to healthcare services, with higher associated maternity costs [7]. It is increasingly recognised that the intrauterine environment, including both poor nutrition and over-nutrition, not only affects pregnancy and neonatal outcomes but also the long term health of the infant [8]; including a higher risk of hypertension in adults born with a LBW [9] and a higher risk of childhood obesity in infants born to women with a high pre-pregnancy weight [10].

Gestational weight gain (GWG) is also an important determinant of pregnancy and birth outcome. Low GWG has been linked to a higher incidence of preterm delivery, LBW and SGA [11]. In contrast excessive GWG has been linked to a higher incidence of CS, induction, maternal weight retention, LGA, macrosomia [11] and obesity development in the offspring [10, 12]. Both inadequate and excessive weight gains in pregnancy have been linked to lower rates of breastfeeding [11]. In view of the many adverse effects of inadequate and excessive weight gain, the Institute of Medicine (IOM) (2009) [13] proposed GWG recommendations dependent upon maternal pre-pregnancy BMI. These recommendations give a range of weight gains in which the likelihood of positive pregnancy outcomes is enhanced [13].

Most of the current evidence on BMI and GWG is from Western or high income countries [11]. Given the importance of maternal pre-pregnancy anthropometric characteristics and GWG on pregnancy and birth outcomes, it is important to explore these factors in communities which are going through a socioeconomic transition with a varied nutritional status across the population. Indonesia in South Asia is such a population. The nutritional status of the population has been captured in recent Indonesian family and life surveys which show the proportion of underweight females (BMI <18.5 kg/m^2^) to have decreased from 17.3% in 1993 to 11.0% in 2007 [14], while the proportion of overweight (BMI between 23.0 and 26.9 kg/m^2^) females has risen from 22.3 to 29.1% and obesity (BMI ≥27.0 kg/m^2^) among women has increased from 9.7 to 19.6% within the same time period. Indonesia is therefore experiencing a nutritional transition, whereby problems and non-communicable diseases, such as obesity and diabetes mellitus are increasing, while infectious diseases and malnutrition remain undefeated [15]. The changing socio-demographic structure in Indonesia is attributable to major shifts in nutrition and overall dietary patterns which have occurred since the remarkable transformation in the Indonesian economy in 1966 [15].

Research into the impact of this shift in dietary and lifestyle pattern among childbearing women in Indonesia is currently limited. Only 2 studies, Achadi et al. (1995) [16] and Winkvist et al. (2002) [17], have examined maternal anthropometric characteristics and GWG in Indonesia. Between these studies women classified as energy deficient decreased from 37.0 to 16.7%, however average GWG remained largely unchanged being 8.9 kg in 1995 and 8.3 kg in 2002. Given the high rate of maternal and infant mortality in Indonesia [18], it is therefore paramount to establish up-to-date baseline information on BMI and GWG and their possible consequences for mothers and babies.

This study was therefore conducted to determine maternal pre-pregnancy BMI and GWG within a pregnant cohort in Western Sumatra, Indonesia and to investigate these on pregnancy and birth outcomes during the study period. The null hypotheses were that BMI has no impact on maternal and infant outcomes and that GWG has no impact on maternal and infant outcomes. We also aimed to compare pregnancy and birth outcomes with regard to nutritional status of the mothers using both International and Asian BMI classifications.

## Methods

In this observational cohort study, data was collected by the midwife caring for any woman who consented to participate, using 3 questionnaires. Questionnaire 1 included socio-demographic factors, obstetric history, pre-pregnancy medical conditions and anthropometric measures. Questionnaire 2 collected data on antenatal outcomes such as the number of antenatal visits. Questionnaire 3 recorded anthropometric measures at three stages during pregnancy; early pregnancy (10–12 weeks), second trimester (22–24 weeks) and third trimester (34–36 weeks). It also included intrapartum, delivery and neonatal outcomes.

### Study setting

The research was carried out in the West Sumatra province of Indonesia. West Sumatra has a total area of 42,013 km^2^ and in 2010 had a population of 4,846,909 [18]. In 2010, West Sumatra had a total fertility rate of 2.9% [19], an infant mortality rate of 47 per 1000 and 89.5% of mothers were attended at their birth by a health professional [18]. Overall Indonesia has a maternal mortality rate of 240 per 100,000 maternities and female life expectancy is 73 years [18].

### Sampling strategy

**Fig. 1:**
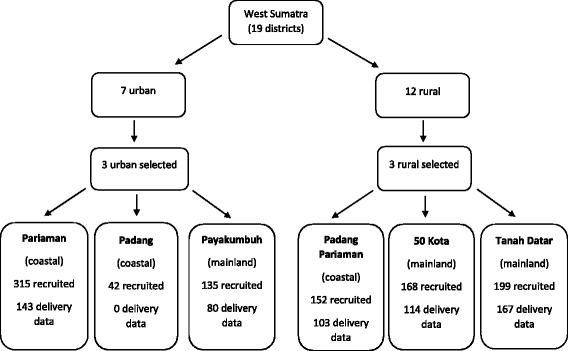
Flow chart showing distirct selection, region classification, recruitment numbers and available delivery data

Stratified random sampling was used to select 3 urban and 3 rural districts to participate within this cohort study, from the 19 districts in West Sumatra – see Fig. 1.

### Recruitment

At least one midwife is placed in every village in West Sumatra. The Midwives Organization within each of the 6 areas was contacted and through collaboration with them, all 537 midwives in the area were recruited to help with the study. This meant every sub-district in each area had a midwife representative. All 537 midwives were trained by the researchers, including explanations about the study aims and objectives and data collection procedures. Women in the first trimester of pregnancy within the identified districts attending their first antenatal visit between August and December 2010 were invited to take part in the study. Study recruitment occurred in government run public health centres, with the exception of Pariaman where some women were recruited from private clinics.

### Standard measures

BMI was calculated from the standard formula weight/height squared (kg/m^2^) using self-reported pre-pregnancy weight. BMI was categorised into underweight, normal-weight, overweight and obese according to both the internationally recognised classifications [20] and the World Health Organization (WHO) recommendations for Asian populations [21] (Table 1). GWG was calculated from self-reported pre-pregnancy weight to the last weight measured by the midwife in the third trimester. The IOM has recommended weight gain ranges for pregnancy according to pre-pregnancy BMI [13] (Table 1). Women were classified as gaining inadequate, recommended or excessive weight during pregnancy in accordance with the international BMI classification and also based on the Asian BMI definitions. Haemoglobin (Hb) <11.0 g/dl was taken as indicative of anaemia as per WHO classifications for pregnant women [22].

**Table 1:** Body mass index and gestational weight gain classifications

	International BMI classification in kg/m^2^ [20]	Asian BMI classification in kg/m^2^ [21]	IOM recommended GWG in kg [13]
Underweight	<18.5	<18.5	12.5–18
Normal	18.5–24.9	18.5–22.9	11.5–16
Overweight	25.0–29.9	23.0–27.4	7–11.5
Obese	≥30.0	≥27.5	5–9

LBW was defined as <2500 g at delivery and macrosomia as >4000 g. Western definitions were used as no Asian standards for birthweight are currently available [23]. Birthweight for gestational age was compared to Alexander et al. (1996) [24] which was the currently accepted standard [25]. Birthweights below the 10th centile were classified as SGA and those above the 90th centile as LGA.

### Data analysis

Logical checks and data cleaning were carried out by the investigators and inconsistencies were returned to the field for clarification. All survey data were double-entered and cleaned using SPSS 24.0. For binary outcomes, logistic regression analyses were used for comparison of groups. Outcomes on a continuous scale were compared using analysis of covariance. Multivariate logistic and linear regression were used to adjust comparisons for confounding factors. For binary outcomes, crude and adjusted odds ratios are reported (OR and aOR) and for continuous outcomes, crude and adjusted mean difference (MD and aMD) are reported, all along with their 95% confidence interval (CI). Confounders included in the adjusted analyses were maternal education, age, parity and district (urban/rural) for BMI comparisons. The same confounders as well as maternal BMI were included in comparisons of gestational weight gain. The OR for perineal suturing was calculated for women achieving a vaginal delivery only. Women with a normal BMI and women with a recommended weight gain were taken as the referent groups. The difference in proportions between the two BMI classification systems were calculated using the two-proportion z-test.

### Ethical approval

This study was conducted according to the guidelines laid down in the Declaration of Helsinki and all procedures involving human subjects/patients were approved by the Faculty of Medicine of Andalas University Ethics Committee (045/KEP/FK/2010). Written informed consent was obtained from all participants.

## Results

The study recruited 1013 women; with 607 of these women having at least partial delivery data available. This article focuses on these 607 women.

### Maternal characteristics

Table 2 presents the characteristics of the 607 study participants with delivery data and the 406 without. Women had a mean age (± standard deviation (SD)) of 28.5 ± 5.6 years, with the majority of the women (82.4%) aged between 20 and 34 years. Of the participating women, 34.5% were nulliparous. None of the women were smokers, all were Minangkabau ethnicity and 74.4% did not have a job outside of the house. The average difference between women’s estimated pre-pregnancy weight and their weight when measured by the midwife in the first trimester was 0.92 kg. Pre-pregnancy estimated weight and actual weight in trimester 1 were highly significantly correlated (Pearson’s correlation 0.930, *p* < 0.001). Average gestational age when women were weighed in trimester 3 was 35.6 (±3.5) weeks. There were no significant differences between participants with and without delivery data for BMI, but there were significant differences in women’s educational level and partner occupation.

**Table 2:** Characteristics of study participants

Maternal characteristics	Participants with delivery data		Participants without delivery data		*P* value
	Mean (±standard deviation)	n	Mean (±standard deviation)	n	
Height (cm)	153.4(±5.6)	581	154.2 (±5.4)	371	0.024
Pre-pregnancy weight (kg)	50.2 (±9.1)	563	50.9 (±9.1)	381	0.270
Pre-pregnancy BMI (kg/m^2^)	21.3 (±3.5)	548	21.3 (±3.5)	353	0.847
Gestational weight gain	10.2 (±6.0)	544	9.3 (±6.5)	21	0.511
Gestational age at delivery (weeks)	39.3 (±2.6)	217	–	–	–
	**N (%)**		**N (%)**		
Maternal age (years):					
< 20	16 (2.7)	603	11 (2.7)	405	0.778
20–24	132 (21.9)		77 (19.0)		
25–29	213 (35.3)		143 (35.3)		
30–34	152 (25.2)		114 (28.1)		
> =35	90 (14.9)		60 (14.8)		
Nulliparous	209 (34.5)	606	139 (34.8)	400	0.932
Educational level - woman:					
Elementary school	101 (16.7)	606	40 (9.9)	404	0.013
Junior High school	141 (23.3)		93 (23.0)		
Senior High School	282 (46.5)		201 (49.8)		
Higher education	82 (13.5)		70 (17.3)		
Occupation^a^ - woman					
Unemployed, student or housewife	445 (74.4)	598	302 (75.5)	400	0.054
Self-employed, trader, services	54 (9.0)		31 (7.8)		
Government employee (civil servant, police, army)	43 (7.2)		41 (10.3)		
Private or state owned enterprise employee	21 (3.5)		16 (4.0)		
Other - ie services, agriculture, labourer	35 (5.9)		10 (2.5)		
Occupation^a^ - partner					
Unemployed, student or housewife	12 (2.0)	599	7 (1.8)	400	0.000
Self-employed, trader, services	412 (68.8)		299 (74.8)		
Government employee (civil servant, police, army)	47 (7.8)		37 (9.3)		
Private or state owned enterprise employee	42 (7.0)		38 (9.5)		
Other - ie agriculture, labourer	86 (14.4)		19 (4.8)		

ANOVA used for continuous data

Chi square used for categorical data

^a^Occupation categorised according to Riset Kesehatan Dasar (RISKESDAS) {Basic Health Research} (2007) [47]

### Maternal pre-pregnancy BMI and its relation to pregnancy and birth outcomes

Within the standard international [20] and Asian specific [21] classification systems 65.3% and 52.9% respectively had a BMI in the normal range (Table 3). On the other hand, 20.1% were underweight and 1.1% and 5.3% were obese respectively using the international and Asian classifications.

**Table 3:** Pregnancy and birth outcomes in relation to pre-pregnancy BMI according to international or Asian classifications

		International BMI category pre-pregnancy			ASIAN BMI category pre-pregnancy		
		<18.5	25.0–29.9	≥30.0	<18.5	23.0–27.4	≥27.5
Numbers in each category (%)		110 (20.1)	74 (13.5)	6 (1.1)	110 (20.1)	119 (21.7)	29 (5.3)
GWG (kg)	MD (95% CI)	2.67 (1.43, 3.92)***	−2.47 (−3.90, −1.04)**	−4.63 (−9.22, −0.03)*	2.21 (0.95, 3.48)**	−2.76 (−3.99, −1.54)***	−3.04 (−5.20, −0.88)**
	aMD (95% CI)‡	2.48 (1.24, 3.73)***	−2.58 (−4.03, −1.13)**	−5.35 (−9.93, −0.78)*	2.07 (0.80, 3.33)**	−2.79 (−4.04, −1.55)***	−3.32 (−5.54, −1.10)**
Birthweight (g)	MD (95% CI)	−19 (−105, 67)	63 (−37, 164)	524 (204, 844)**	11 (−77, 99)	129 (43, 214)**	196 (42, 350)*
	aMD (95% CI)‡	−15 (−102, 72)	53 (−50, 156)	556 (234, 878)**	10 (−79, 99)	117 (29, 205)**	205 (46, 365)*
Number of antenatal visits	MD (95% CI)	0.27 (−0.52, 1.05)	1.06 (0.13, 2.00)*	−1.23 (−4.13, 1.68)	0.44 (−0.37, 1.25)	1.14 (0.34, 1.94)**	0.51 (−0.94, 1.96)
	aMD (95% CI)‡	0.30 (−0.48, 1.08)	0.81 (−0.13, 1.74)	−1.83 (−4.69, 1.04)	0.49 (−0.31, 1.28)	1.10 (0.30, 1.90)**	0.20 (−1.27, 1.67)
Gestation at delivery (weeks)	MD (95% CI)	0.24 (−0.69, 1.17)	0.55 (−0.53, 1.63)	1.17 (−1.87, 4.21)	0.18 (−0.77, 1.12)	0.13 (−0.91, 1.17)	0.39 (−1.10, 1.87)
	aMD (95% CI)‡	0.15 (−0.82, 1.11)	0.71 (−0.42, 1.85)	1.04 (−2.12, 4.19)	0.90 (−0.89, 1.07)	0.25 (−0.82, 1.32)	0.55 (−1.04, 2.13)
Inadequate weight gain	OR (95% CI)	0.53 (0.34, 0.83)**	0.36 (0.21, 0.60)***	0.29 (0.05, 1.62)	0.58 (0.37, 0.91)*	0.32 (0.20, 0.50)***	0.29 (0.13, 0.65)**
	aOR (95% CI)‡	0.56 (0.36, 0.88)*	0.37 (0.22, 0.63)***	0.33 (0.06, 1.86)	0.61 (0.39, 0.96)*	0.31 (0.20, 0.50)***	0.31 (0.13, 0.72)**
Trimester 2 Haemoglobin <11.0g/dl	OR (95% CI)	1.27 (0.78, 2.05)	0.54 (0.29, 1.01)	0.84 (0.14, 5.02)	1.17 (0.71, 1.91)	0.55 (0.33, 0.92)*	0.61 (0.24, 1.60)
	aOR (95% CI)‡	1.32 (0.80, 2.17)	0.47 (0.24, 0.90)*	0.89 (0.14, 5.79)	1.18 (0.71, 1.97)	0.45 (0.26, 0.79)**	0.55 (0.19, 1.54)
Trimester 3 Haemoglobin <11.0 g/dl	OR (95% CI)	1.11 (0.66, 1.86)	0.71 (0.38, 1.36)	1.27 (0.21, 7.73)	1.08 (0.63, 1.84)	0.84 (0.50, 1.41)	0.62 (0.22, 1.77)
	aOR (95% CI)‡	1.18 (0.69, 2.00)	0.75 (0.38, 1.45)	1.60 (0.25, 9.99)	1.12 (0.65, 1.93)	0.75 (0.43, 1.29)	0.76 (0.25, 2.27)
Induction	OR (95% CI)	1.17 (0.50, 2.69)	1.86 (0.82, 4.22)	2.95 (0.32, 27.43)	1.24 (0.52, 2.95)	1.50 (0.70, 3.23)	2.63 (0.81, 8.57)
	aOR (95% CI)‡	1.06 (0.45, 2.51)	1.92 (0.81, 4.58)	1.71 (0.18, 16.53)	1.17 (0.48, 2.86)	1.78 (0.79, 4.02)	2.31 (0.65, 8.12)
Spontaneous vaginal delivery	OR (95% CI)	1.32 (0.71, 2.48)	0.55 (0.30, 0.99)*	0.40 (0.07, 2.25)	1.33 (0.70, 2.53)	0.77 (0.45, 1.33)	0.53 (0.22, 1.27)
	aOR (95% CI)‡	1.55 (0.81, 2.99)	0.53 (0.28, 1.02)	0.51 (0.09, 3.00)	1.51 (0.78, 2.95)	0.63 (0.35, 1.14)	0.64 (0.24, 1.68)
Caesarean section	OR (95% CI)	0.76 (0.38, 1.52)	1.70 (0.89, 3.23)	3.28 (0.58, 18.39)	0.79 (0.39, 1.60)	1.32 (0.72, 2.40)	2.58 (1.07, 6.26)*
	aOR (95% CI)‡	0.68 (0.33, 1.41)	1.55 (0.77, 3.11)	2.61 (0.43, 15.98)	0.72 (0.34, 1.50)	1.43 (0.75, 2.72)	2.05 (0.77, 5.51)
LBW <2.5 kg	OR (95% CI)	1.89 (0.54, 6.57)	2.86 (0.81, 10.03)	–	1.52 (0.44, 5.30)	1.04 (0.26, 4.09)	1.45 (0.17, 12.23)
	aOR (95% CI)‡	1.77 (0.98, 6.27)	3.14 (0.84, 11.65)	–	1.47 (0.42, 5.21)	1.19 (0.29, 4.89)	1.29 (0.14, 11.89)
Macrosomia >4.0 kg	OR (95% CI)	0.65 (0.08, 5.58)	1.95 (0.37, 10.28)	34.20 (5.05, 231.71)***	0.87 (0.09, 8.50)	2.46 (0.49, 12.37)	11.12 (2.13, 58.01)**
	aOR (95% CI)‡	0.66 (0.07, 5.80)	1.67 (0.30, 9.11)	104.84 (6.15, 1788.21)**	0.91 (0.09, 8.91)	2.54 (0.48, 13.47)	13.46 (2.32, 77.99)**
SGA	OR (95% CI)	0.88 (0.30, 2.60)	0.75 (0.20, 2.80)	–	0.76 (0.26, 2.24)	0.18 (0.02, 1.43)	0.93 (0.19, 4.60)
	aOR (95% CI)‡	0.68 (0.21, 2.18)	0.62 (0.14, 2.69)	–	0.62 (0.20, 2.00)	0.23 (0.03, 1.87)	0.60 (0.09, 3.88)
LGA	OR (95% CI)	0.89 (0.09, 8.84)	5.87 (1.23, 27.88)*	–	1.18 (0.10, 13.41)	5.02 (0.80, 31.49)	8.08 (1.04, 62.77)*
	aOR (95% CI)‡	1.56 (0.13, 18.48)	5.50 (0.96, 31.50)	–	2.07 (0.15, 27.89)	4.81 (0.72, 32.07)	7.10 (0.65, 77.93)
Born <37 + 0 weeks	OR (95% CI)	0.69 (0.27, 1.73)	0.26 (0.06, 1.18)	–	0.68 (0.27, 1.72)	0.48 (0.15, 1.51)	0.27 (0.03, 2.16)
	aOR (95% CI)‡	0.73 (0.28, 1.89)	0.22 (0.05, 1.01)	–	0.72 (0.28, 1.90)	0.44 (0.14, 1.40)	0.23 (0.03, 2.01)
Born >41 + 6 weeks	OR (95% CI)	0.65 (0.21, 2.08)	0.74 (0.20, 2.72)	–	0.60 (0.19, 1.93)	0.59 (0.16, 2.17)	0.45 (0.06, 3.71)
	aOR (95% CI)‡	0.59 (0.18, 1.96)	0.80 (0.21, 3.14)	–	0.56 (0.17, 1.85)	0.60 (0.16, 2.29)	0.51 (0.06, 4.56)
Postpartum haemorrhage	OR (95% CI)	0.86 (0.32, 2.36)	1.73 (0.70, 4.28)	3.07 (0.34, 27.55)	1.00 (0.35, 2.84)	2.11 (0.94, 4.71)	1.42 (0.31, 6.60)
	aOR (95% CI)‡	0.87 (0.31, 2.42)	1.89 (0.74, 4.85)	3.35 (0.36, 31.61)	0.97 (0.34, 2.80)	1.93 (0.84, 4.47)	1.81 (0.37, 8.92)
Perineal sutures	OR (95% CI)	1.47 (0.92, 2.37)	0.79 (0.45, 1.41)	3.17 (0.33, 30.81)	1.27 (0.78, 2.07)	0.57 (0.35, 0.93)*	0.66 (0.26, 1.71)
	aOR (95% CI)‡	1.11 (0.66, 1.89)	1.13 (0.59, 2.19)	1.88 (0.19, 18.87)	1.07 (0.62, 1.83)	0.95 (0.55, 1.65)	0.91 (0.30, 2.74)
Initial feed at breast	OR (95% CI)	1.31 (0.43, 3.98)	0.22 (0.10, 0.46)***	0.08(0.01, 0.49)**	1.24 (0.39, 3.88)	0.33 (0.15, 0.72)**	0.22 (0.07, 0.68)**
	aOR (95% CI)‡	1.34 (0.43, 4.15)	0.25 (0.12, 0.56)**	0.15 (0.02, 1.00)	1.26 (0.40, 4.04)	0.31 (0.14, 0.70)**	0.33 (0.10, 1.08)
Breastfeeding at discharge	OR (95% CI)	1.07 (0.61, 1.89)	0.49 (0.28, 0.85)*	0.37 (0.06, 2.27)	1.06 (0.59, 1.89)	0.66 (0.40, 1.10)	0.52 (0.22, 1.20)
	aOR (95% CI)‡	1.04 (0.59, 1.85)	0.52 (0.29, 0.92)*	0.47 (0.08, 2.95)	1.03 (0.57, 1.85)	0.67 (0.39, 1.13)	0.58 (0.24, 1.39)
Back pain	OR (95% CI)	1.15 (0.36, 3.65)	1.24 (0.34, 4.51)	–	1.11 (0.34, 3.62)	1.20 (0.40, 3.60)	–
	aOR (95% CI)‡	1.21 (0.36, 4.07)	1.06 (0.28, 4.07)	–	1.16 (0.34, 3.97)	1.02 (0.31, 3.34)	–
Neonatal intensive care admission	OR (95% CI)	–	2.55 (0.46, 14.22)	19.75 (1.79, 218.36)*	–	0.59 (0.07, 5.30)	6.02 (1.04, 34.83)*
	aOR (95% CI)‡	–	1.89 (0.31, 11.74)	12.90 (1.01, 164.98)*	–	0.53 (0.06, 5.21)	3.93 (0.53, 29.19)

Reference group: normal BMI 18.5–24.9 kg/m^2^ within international classification and 18.5–22.9 kg/m^2^ within Asian BMI classification

‡Adjusted for woman’s education, district type (urban/ rural), maternal age and parity

*BMI* Body mass index, *n* number, *GWG* gestational weight gain, *MD* mean difference, *aMD* adjusted mean difference, *OR* odds ratio, *aOR* adjusted odds ratio, *CI* confidence interval, *LBW* low birth weight, *SGA* small for gestational age, *LGA* large for gestational age

* = *p* < 0.05, ** = *p* < 0.01, *** = *p* < 0.001

Pregnancy and birth outcome proportions according to pre-pregnancy BMI are presented in Additional file 1. Within this cohort, mean maternal GWG ± SD was 10.2 ± 6.0 kg (*n* = 544) and mean birthweight was 3165 ± 402 g (*n* = 577). The results of the regression and multivariate analyses for maternal and neonatal outcomes are shown in Table 3 for both international [20] and Asian [21] BMI classifications. GWG was significantly higher in the underweight group, adjusted mean difference (aMD) (95% CI) 2.48 (1.24 to 3.73)kg for international and aMD 2.07 (0.80 to 3.33)kg for Asian BMI classification compared to women with a BMI in the normal range. Weight gain during pregnancy was reduced for women who were overweight and obese compared to women with a normal BMI within both BMI classifications. However when classifying GWG according to IOM recommendations, the odds of inadequate weight gain were lower in underweight, overweight and obese women than in women of normal weight, however this was not significant in the obese subgroup when using international BMI classifications. Mean birthweight was significantly increased in women who were obese within the international classification aMD 556 (234–878)g and in both the overweight and obese categories using the Asian BMI classification, aMD 117 (29–205)g and aMD 205 (46–365)g respectively.

As evident in Table 3, women who were obese had higher odds of having a macrosomic infant compared to the reference group of normal weight women. Women who were overweight had lower adjusted odds for giving their baby an initial breastfeed after delivery in both classification systems; adjusted odds ratio (95% CI) aOR 0.25 (0.12 to 0.56) for the international BMI classification and 0.31 (0.14 to 0.70) for Asian BMI classification. When using international BMI classifications, women with a high BMI also had lower odds of exclusively breastfeeding at discharge from hospital aOR 0.52 (0.29 to 0.92) and had higher odds of having an infant admitted to a neonatal intensive care unit (NICU) aOR 12.90 (1.01 to 164.98). When using the Asian BMI classification, women who were overweight had lower odds of haemoglobin less than 11.0 g/dl in trimester 2 aOR 0.45 (0.26 to 0.79). It was not possible to analyse the incidence of shoulder dystocia, APGAR score < 7 at 5 min, postnatal depression, Sudden infant death syndrome, and maternal mortality across the different BMI or weight gain categories as there were too few cases in the whole sample.

### Gestational weight gain based on IOM recommendations and its relationship with pregnancy and birth outcomes

**Fig. 2:**
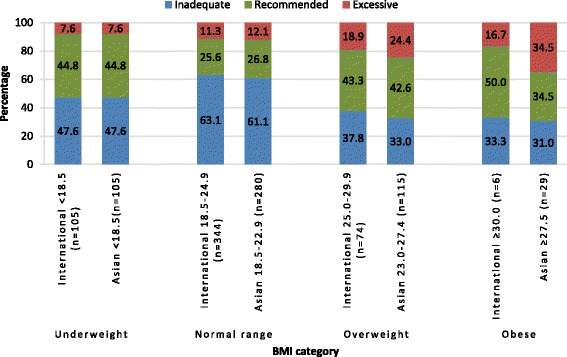
Weight gain according to the Institute of Medicine recommendations applied to international and Asian body mass index classifications (*n* = 529)

Irrespective of BMI classification system, a large proportion of women who were underweight (47.6%) or had a BMI in the normal range prior to pregnancy (>60%) gained inadequate weight according to IOM guidance during pregnancy (Fig. 2). In contrast for overweight or obese women, more gained the recommended amount of weight than inadequate weight. Over all BMI categories, when IOM recommendations were adapted to Asian BMI classifications, 50.7% of women gained inadequate weight during pregnancy, compared to 56.1% using international classifications. The difference in the proportion of women gaining inadequate weight using the two different BMI classifications was not quite significant (*p* = 0.073).

Pregnancy and birth outcome proportions according to IOM weight gain category are presented in Additional file 2 and results of the regression and multivariate analyses for maternal and neonatal outcomes according to weight gain classification are shown in Table 4. When adjusted for confounding factors (maternal age, pre-pregnancy BMI, parity, level of education and district (urban/rural)), significant reductions were observed in birthweight aMD (95% CI) -139 (−215, −64) and gestational age at delivery −1.18 (−2.02, −0.35) and also in the odds of macrosomia aOR (95% CI) 0.10 (0.01, 0.87), post-term birth aOR 0.34 (0.13, 0.93), perineal sutures aOR 0.51 (0.31, 0.83) and back pain aOR 0.07 (0.10, 0.60) and increased odds of SGA aOR 5.44 (1.36, 21.77) and preterm birth aOR 3.55 (1.23, 10.21) with inadequate GWG compared to recommended weight gain, when applying IOM weight gain recommendations to the Asian BMI classification system. Significant differences were also identified when applying IOM weight gain recommendations to the international BMI classification system in all of these outcomes except for the lower adjusted odds of having a macrosomic baby with inadequate GWG.

**Table 4:** Pregnancy and birth outcomes in relation to gestational weight gain according to IOM recommendations based on international and Asian BMI classifications

		IOM weight gain recommendations applied to international BMI classification		IOM weight gain recommendations applied to Asian BMI classification	
		Inadequate	Excessive	Inadequate	Excessive
Number in each category (%)		297 (56.1%)	62 (11.7%)	268 (50.7%)	80 (15.1%)
Mean birthweight (g)	MD (95% CI)	−98 (−173, −22)*	131 (14, 248)*	−139 (−215, −64)***	83 (−23, 188)
	aMD (95% CI)‡	−104 (−180, −29)**	128 (12, 245)*	−139 (−215, −64)***	63 (−43, 169)
Number of antenatal visits	MD (95% CI)	−0.27 (−0.97, 0.44)	0.62 (−0.46, 1.71)	−0.48 (−1.18, 0.23)	0.62 (−0.37, 1.60)
	aMD (95% CI)‡	−0.20 (−0.89, 0.50)	0.45 (−0.63, 1.52)	−0.43 (−1.13, 0.27)	0.43 (−0.64, 1.41)
Gestation at delivery	MD (95% CI)	−1.22 (−2.03, −0.42)**	−0.45 (−1.77, 0.88)	−1.13 (−1.95, −0.31)**	−0.38 (−1.56, 0.80)
	aMD (95% CI)‡	−1.28 (−2.10, −0.45)**	−0.42 (−1.78, 0.94)	−1.18 (−2.02, −0.35)**	−0.37 (−1.63, 0.89)
Haemoglobin <11.0 g/dl in trimester 2	OR (95% CI)	1.26 (0.82, 1.94)	1.09 (0.56, 2.10)	1.23 (0.80, 1.90)	0.98 (0.54, 1.80)
	aOR (95% CI) ‡	1.28 (0.81, 2.01)	1.39 (0.69, 2.79)	1.22 (0.77, 1.93)	1.23 (0.65, 2.35)
Haemoglobin <11.0 g/dl in trimester 3	OR (95% CI)	1.42 (0.90, 2.25)	1.19 (0.59, 2.42)	1.46 (0.92, 2.32)	1.17 (0.61, 2.23)
	aOR (95% CI) ‡	1.41 (0.88, 2.26)	1.32 (0.64, 2.74)	1.45 (0.91, 2.32)	1.31 (0.67, 2.56)
Induction	OR (95% CI)	0.69 (0.35, 1.40)	1.34 (0.52, 3.48)	0.81 (0.39, 1.66)	1.75 (0.74, 4.12)
	aOR (95% CI) ‡	0.73 (0.35, 1.50)	1.13 (0.42, 3.06)	0.85 (0.40, 1.79)	1.60 (0.65,3.92)
Spontaneous vaginal delivery	OR (95% CI)	1.20 (0.73, 1.97)	0.98 (0.47, 2.04)	1.23 (0.74, 2.02)	0.92 (0.47, 1.78)
	aOR (95% CI)‡	1.13 (0.67, 1.91)	1.15 (0.52, 2.51)	1.10 (0.65, 1.85)	1.06 (0.52, 2.17)
Caesarean section	OR (95% CI)	0.67 (0.39, 1.13)	0.72 (0.31, 1.67)	0.66 (0.38, 1.14)	0.90 (0.44, 1.87)
	aOR (95% CI)‡	0.69 (0.39, 1.20)	0.59 (0.24, 1.43)	0.72 (0.41, 1.27)	0.75 (0.34, 1.67)
LBW <2.5 kg	OR (95% CI)	0.89 (0.31, 2.53)	–	1.24 (0.41, 3.76)	0.45 (0.05, 3.89)
	aOR (95% CI)‡	0.87 (0.30, 2.51)	–	1.25 (0.41, 3.86)	0.47 (0.05, 4.21)
Macrosomia >4.0 kg	OR (95% CI)	0.19 (0.04, 0.96)*	0.94 (0.18, 4.77)	0.11 (0.01, 0.93)*	1.14 (0.28, 4.68)
	aOR (95% CI)‡	0.19 (0.04, 1.02)	1.04 (0.18, 5.95)	0.10 (0.01, 0.87)*	0.85 (0.19, 3.86)
SGA	OR (95% CI)	2.79 (0.89, 8.69)	0.77 (0.08, 7.32)	3.87 (1.09, 13.82)*	1.48 (0.23, 9.46)
	aOR (95% CI)‡	4.52 (1.26, 16.24)*	0.72 (0.07, 7.78)	5.44 (1.36, 21.77)*	1.22 (0.14, 10.31)
LGA	OR (95% CI)	0.35 (0.06, 2.18)	2.21 (0.34, 14.39)	1.12 (0.10, 12.60)	10.10 (1.07, 95.70)*
	aOR (95% CI)‡	0.29 (0.04, 2.27)	2.25 (0.29, 17.50)	0.68 (0.05, 8.97)	5.66 (0.48, 66.64)
Born <37 weeks	OR (95% CI)	3.17 (1.14, 8.80)*	2.03 (0.44, 9.41)	3.18 (1.14, 8.88)*	1.94 (0.48, 7.95)
	aOR (95% CI)‡	3.38 (1.17, 9.73)*	2.76 (0.55, 13.69)	3.55 (1.23, 10.21)*	3.14 (0.70, 14.19)
Born ≥42 weeks	OR (95% CI)	0.30 (0.12, 0.79)*	0.47 (0.09, 2.33)	0.35 (0.13, 0.94)*	0.56 (0.14, 2.21)
	aOR (95% CI)‡	0.27 (0.10, 0.73)*	0.58 (0.11, 3.02)	0.34 (0.13, 0.93)*	0.82 (0.19, 3.55)
Postpartum Haemorrhage	OR (95% CI)	1.00 (0.43, 2.33)	2.26 (0.80, 6.37)	1.03 (0.43, 2.47)	2.45 (0.93, 6.44)
	aOR (95% CI)‡	0.98 (0.41, 2.34)	2.11 (0.72, 6.17)	1.13 (0.47, 2.77)	2.40 (0.88, 6.51)
Perineal sutures	OR (95% CI)	0.60 (0.39, 0.91)*	0.85 (0.44, 1.64)	0.60 (0.39, 0.91)*	0.63 (0.34, 1.14)
	aOR (95% CI)‡	0.56 (0.34, 0.91)*	0.72 (0.34, 1.50)	0.51 (0.31, 0.83)**	0.57 (0.29, 1.14)
Initial feed at breast	OR (95% CI)	1.89 (0.91, 3.93)	0.97 (0.36, 2.59)	1.59 (0.75, 3.38)	0.82 (0.33, 2.01)
	aOR (95% CI)‡	1.68 (0.78, 3.62)	1.16 (0.41, 3.32)	1.33 (0.61, 2.92)	1.05 (0.40, 2.78)
Breastfeeding at discharge	OR (95% CI)	0.86 (0.54, 1.38)	0.95 (0.46,1.94)	0.83 (0.52, 1.32)	1.02 (0.53, 1.96)
	aOR (95% CI)‡	0.86 (0.54, 1.39)	1.02 (0.49, 2.13)	0.83 (0.51, 1.33)	1.20 (0.61, 2.36)
Back pain	OR (95% CI)	0.05 (0.01, 0.37)**	1.43 (0.51, 4.01)	0.07 (0.01, 0.54)*	1.96 (0.74, 5.16)
	aOR (95% CI)‡	0.06 (0.01, 0.43)**	1.29 (0.42, 3.96)	0.07 (0.10, 0.60)*	2.10 (0.70, 6.25)
Neonatal intensive care admission	OR (95% CI)	0.41 (0.07, 2.49)	1.94 (0.32, 11.91)	0.74 (0.10, 5.28)	3.64 (0.60, 22.27)
	aOR (95% CI)‡	0.50 (0.08, 3.19)	1.44 (0.22, 9.65)	0.89 (0.12, 6.68)	2.40 (0.35, 16.33)

Reference group: recommended weight gain

‡Adjusted for woman’s education, district type (urban/ rural), maternal age, parity and maternal body mass index

*IOM* institute of medicine, *BMI* Body mass index, *MD* mean difference, *aMD* adjusted mean difference, *OR* odds ratio, *aOR* adjusted odds ratio, *LBW* low birth weight, *SGA* small for gestational age, *LGA* large for gestational age

* = *p* < 0.05, ** = *p* < 0.01, *** = *p* < 0.001

## Discussion

Pre-pregnancy BMI and weight gain during pregnancy reflect maternal nutritional status both before and during pregnancy and are an indicator of reserves for fetal growth. In this cohort of Indonesian pregnant women from West Sumatra, a considerable proportion of women were underweight (20.1%) and a much lower percentages were overweight and obese (ranging from 14.6–27.0% using international or Asian BMI classification systems respectively). Overall, inadequate GWG was observed among more than half of all pregnant women in this study. Looking at differences between BMI categories using both classification systems, inadequate GWG was highest in normal-weight women (>60%), followed by under-weight, overweight and obese groups respectively. Significant adverse pregnancy and birth outcomes were associated with inadequate GWG and in women of low or high BMI categories. Adverse outcomes such as preterm birth and SGA associated with inadequate GWG in this study, are of utmost significance considering the high incidence of maternal and perinatal mortality in this population [18]. Prematurity has been considered a major killer factor contributing to infant neonatal mortality in developed countries [26]. In line with the global strategy for Women’s, children’s and adolescents’ health and wellbeing in support of sustainable development goals, promoting principles of survive, thrive and transform, our study reinforces the importance of giving attention to enhancing maternal nutrition in order to reduce health inequalities for mothers and their babies [27]. These results highlight the need for urgent actions to identify appropriate interventions to enhance the nutritional status of pregnant mothers in Indonesia both in terms of pre-pregnancy BMI status and gestational weight gain.

### National and international context

The prevalence of women with a BMI of 25 kg/m^2^ or greater was higher than in the study by Winkvist et al. (2002) a decade before in Java; however the level of under nutrition (BMI <18.5 kg/m^2^) prior to pregnancy was also higher [17]. This may therefore indicate that societal transition is leading to an exacerbated situation in terms of diversity in nutritional health in Indonesia; so that while still struggling with under-nutrition, they are also facing the challenge of overweight. Within this study a high proportion of women did not achieve the recommended weight gain during pregnancy, particularly women with a low or normal BMI. However the mean GWG and the proportion of women gaining the recommended amount of weight in this cohort was larger than in previous Indonesian studies in the coastal region of West Java in 1995 [16] and in a mainly rural area of Central Java in 2002 [17]. Recommended weight gain in this study was in line with other recent studies conducted in middle income countries including Pakistan [28] and Iran [29], however adherence was markedly different from high income country studies such as the US, Canada and Sweden where the proportion of women with excessive GWG is far higher [30–34]. The high incidence of inadequate GWG is of particular importance for clinicians, researchers and policy makers to develop and adapt strategic interventions in order to address these modifiable nutritional deficiencies which are known to have a significant impact on infant weight and wellbeing [11].

### Birthweight outcomes

Birthweight was significantly higher in women who were obese and was significantly lower in those gaining inadequate weight within both BMI classifications. The odds of macrosomia was higher in women who were obese. The crude odds of LGA were also higher in overweight women with international BMI classification and in women in the obese category using the Asian BMI classification, however once adjusting for confounders these were no longer significant. This is in line with previous research which has shown increased pre-pregnancy BMI to be associated with an higher incidence of LGA and macrosomia using both international [28, 31, 35–37] and Asian BMI classifications [38–40]. When comparing IOM weight gain groups (inadequate, recommended, excessive) (Table 4) applied to Asian BMI classifications the odds of macrosomia was significantly lower in women gaining inadequate weight and the crude odds of LGA were higher in those gaining excessive weight, however once controlling for other factors this was no longer significant. The odds of SGA were also higher in women gaining inadequate weight using both international and Asian BMI classifications. There is much evidence that weight gain below that recommended by the IOM is associated with LBW [11, 41] and SGA infants [11, 31, 33, 36, 41] and exceeding weight gain recommendations is associated with higher proportions of macrosomia [41] and LGA infants [11, 31, 36, 37, 41]. Given that macrosomia is associated with a higher risk of mortality and morbidity [42] and LBW and SGA with neonatal mortality and chronic diseases later in life such as glucose intolerance, coronary heart disease, obesity and disturbed blood clotting [43]; ensuring adequate maternal nutrition and weight gain in pregnancy in order to improve birth outcomes is of paramount importance.

### Other outcomes

Compared to women of a normal BMI, the odds of anaemia (haemoglobin level < 11.0 g/dl) were lower in overweight women when first tested in pregnancy in trimester 2. This may have been one of the mediating factors for higher birthweight with increasing BMI within this cohort, as maternal anaemia is known to influence birthweight outcomes [44]. When haemoglobin levels were measured in the 3rd trimester a lower incidence of anaemia was noted (Additional file 1) and there were no differences in odds between BMI categories. This may be due to the Indonesian policy of distributing iron supplementation tablets to all pregnant mothers. Given the known association between adverse outcomes and anaemia [44] and the large proportion of women within our study who were anaemic in early pregnancy, supplementation of Indonesian women prior to pregnancy seems appropriate. Any measures to optimise maternal BMI for pregnancy in Indonesia will need to address a potential resultant increased vulnerability to anaemia. Furthermore, our finding of a high incidence of anaemia and under-nutrition in this population may indicate insufficiency in other macro/micronutrients [45] which were outside the scope of this study.

There were lower odds of breastfeeding initiation at delivery for women who were overweight or obese compared to those of normal BMI, although once adjusting for other factors this only remained significant in the overweight group. The reduction in breastfeeding in higher BMIs in our study is of interest as it is consistent with global evidence [6, 11, 46]. Many varied reasons have been offered for these differences including physiological, psychological and socio-cultural factors, as well as the increase of co-existing medical pathologies alongside their associated higher risk of assisted birth that occurs with obesity [6]. The crude odds of CS were higher in obese women compared to women with a normal BMI, which is consistent with the literature [35, 36].

### Comparison of Asian specific and international BMI classification systems

Although there is an ongoing debate about the appropriateness of Asian or International BMI classifications for specific communities, due to a high correlation between BMI, body fat percentage and health risks, WHO has suggested the use of specific BMI categories for Asian populations [21]. Evidence on adverse pregnancy outcomes and BMI cut off points for Asian populations, are limited. Assessing accuracy or comparing predictability or appropriateness of each BMI classification system for the pregnant women was not a primary objective of this study. As a side observation we have found similar results in observing significant associations between adverse pregnancy outcomes using international and Asian classifications in this population, however the Asian criteria seems to be more sensitive in identifying adverse outcomes. Encouraging women to gain weight in accordance with the IOM recommendations adapted for ethnicity could maximise the outcome of pregnancy in this group of women.

### Strengths and limitations

The strengths of this research were that data collection was prospective and that it incorporated women from diverse regions of West Sumatra, including urban, rural, mainland and coastal areas, with a widespread engagement of midwives. A heterogeneous sample was obtained, as ascertained from maternal and partner occupation and education levels.

This is one of the few studies attempting to use both international and Asian specific BMI classifications in addressing the interrelationship among maternal nutritional status via anthropometric characteristics and weight gain during pregnancy. This large study, is of significance addressing global priority areas for women and their babies in a developing country with considerable societal and economic transitions.

A limitation of the study was that delivery data was only available for 60% of those initially recruited. However when we looked at demographic data for participants without delivery data compared to those with delivery data, there were no significant differences between the 2 groups except for the included women having significantly lower education levels and their partners occupation being more likely to be in agriculture or a labourer. Even when interpreting the results in the context of these differences, they still demonstrate high proportions of under nutrition and inadequate weight gain in these women. Delivery data only being available for 60% of the women recruited also led to small numbers within some categories, which may have reduced the power of calculating statistical differences and the number of outcomes that could be analysed. However the sample size has been adequate to demonstrate statistically significant differences in main outcomes such as birthweight, SGA and preterm birth.

It was difficult to ascertain the representativeness of our study sample to the actual population, due to the lack of available information on maternal age and parity in this region and due to numerous classification systems for occupation being in place within the official regional statistics. Our sample did however appear to be slightly more educated than the population in general and due to the stratified sampling technique our sample had more women living in urban and coastal areas than the average across the region.

A further limitation of the study is that pre-pregnancy BMI was calculated from women’s self-reported pre-pregnancy weight, however this is common within the literature in this area [4, 12, 23]. Furthermore within this study average self-reported pre-pregnancy weight and average actual weight in trimester 1 only differed by 0.9 kg and there was a high correlation between the two variables suggesting that biases from using estimated pre-pregnancy weight would be minimal.

## Conclusion

Many women in West Sumatra embark on pregnancy with a suboptimal BMI and the majority gain inadequate weight according to IOM recommendations. This was irrespective of whether BMI was calculated using the international or Asian BMI classifications. Weight gain decreased with increasing pre-pregnancy BMI, while the odds of macrosomia were higher. Inadequate GWG was related to poor pregnancy outcomes, including SGA and prematurity.

These findings are of paramount significance for the attention of researchers, policy makers and decision-making organisations to facilitate development of culturally sensitive interventions to enhance nutritional status and health of mothers and babies, in an area known for its high incidence of maternal and neonatal mortality.

Further investigation to identify the magnitude and interaction among maternal nutritional status, education, urbanisation, environment, access to food and socioeconomic factors and neonatal birthweight, health and wellbeing are required. The safety of weight gain limitations in women according to pre-pregnancy BMI category and also in relation to anaemia and other nutritional deficiencies is needed, particularly in developing countries.

## Additional files

Additional file 1: Comparison of pregnancy and birth outcomes according to pre-pregnancy BMI categories using international and Asian classifications. This gives a table of pregnancy and birth outcome proportions according to pre-pregnancy BMI for the two BMI classification systems. (PDF 45 kb)

Additional file 2: Pregnancy and birth outcomes in relation to various gestational weight gains according to IOM recommendations for all BMI groups combined based on international and Asian classifications. This gives a table of pregnancy and birth outcome proportions according to IOM weight gain category, when applied to each BMI classification system. (PDF 38 kb)

## Abbreviations

aMD: Adjusted mean difference
AN: Antenatal
aOR: Adjusted odds ratio
BMI: Body mass index
CI: Confidence interval
CS: Caesarean section
GWG: Gestational weight gain
IOM: Institute of Medicine
LGA: Large for gestational age
MD: Mean difference
NICU: Neonatal intensive care unity
OR: Odds ratio
SD: Standard deviation
SGA: Small for gestational age
WHO: World Health Organization
