# Supplementary material for: Pre-pregnancy body mass index and gestational weight gain and their effects on pregnancy and birth outcomes: a cohort study in West Sumatra, Indonesia
Source: BMC Womens Health. 2017 Nov 9;17:102. doi: 10.1186/s12905-017-0455-2 (PMC5679340; doi:10.1186/s12905-017-0455-2)
Supplement: Supplementary file 1 — Comparison of pregnancy and birth outcomes according to pre-pregnancy BMI categories using international and Asian classifications. This gives a table of pregnancy and birth outcome proportions according to pre-pregnancy BMI for the two BMI classification systems. (PDF 45 kb) [file 12905_2017_455_MOESM1_ESM.pdf]

Comparison of pregnancy and birth outcomes according to pre-pregnancy BMI categories using international and Asian classifications

	Sample proportion/ average	**International BMI category pre-pregnancy**					**ASIAN BMI category pre-pregnancy**				
		**<18.5**	**18.5-24.9**	**25.0-29.9**	**≥30.0**	**Overall p value**	**<18.5**	**18.5-22.9**	**23.0-27.4**	**≥27.5**	**Overall p value**
Mean GWG (kg)<br>SD<br>n	10.2<br>6.0<br>544	12.7<br>4.7<br>105	10.0<br>5.7<br>344	7.6<br>6.6<br>74	5.4<br>5.9<br>6	**0.000*** **	12.7<br>4.7<br>105	10.5<br>5.2<br>280	7.7<br>6.9<br>115	7.5<br>6.8<br>29	**0.000*** **
Mean birthweight (g)<br>SD<br>n	3165<br>402<br>577	3140<br>370<br>107	3159<br>382<br>347	3222<br>479<br>72	3683<br>519<br>6	**‡ 0.040***	3140<br>370<br>107	3129<br>380<br>281	3258<br>433<br>116	3325<br>462<br>28	**‡0.007*** **
Mean number of AN visits<br>SD<br>n	10.0<br>3.6<br>565	10.2<br>3.8<br>106	9.9<br>3.4<br>331	11.0<br>4.1<br>69	8.7<br>3.4<br>6	**‡ 0.161**	10.2<br>3.8<br>106	9.7<br>3.3<br>272	10.9<br>4.0<br>108	10.2<br>4.4<br>26	**‡ 0.069**
Mean gestation at delivery (Weeks)<br>SD<br>n	39.3<br>2.6<br>217	39.4<br>2.3<br>43	39.2<br>2.9<br>118	39.7<br>1.9<br>29	40.3<br>1.3<br>3	**‡ 0.830**	39.4<br>2.3<br>43	39.2<br>2.8<br>103	39.4<br>2.8<br>33	39.6<br>1.6<br>14	**‡ 0.964**
Inadequate weight gain	Int 297/529 (56.1%)<br>Asian 268/597 (50.7%)	50/105 (47.6%)	217/344 (63.1%)	28/74 (37.8%)	2/6 (33.3%)	**§0.000*** **	50/105 (47.6%)	171/280 (61.1%)	38/115 (33.0%)	9/29 (31.0%)	**0.000*** **
Trimester 2 Haemoglobin <11.0g/dl	213/473 (45.0%)	44/87 (50.6%)	125/280 (44.6%)	16/53 (30.2%)	2/5 (40.0%)	**§0.114**	44/87 (50.6%)	107/229 (46.7%)	29/89 (32.6%)	7/20 (35.0%)	**0.056**
Trimester 3 Haemoglobin <11.0g/dl	157/464 (33.8%)	29/79 (36.7%)	95/276 (34.4%)	15/55 (27.3%)	2/5 (40.0%)	**§0.681**	29/79 (36.7%)	78/223 (35.0 %)	29/93 (31.2%)	5/20 (25.0%)	**0.704**
Induction	44/511 (8.6%)	8/89 (9.0%)	24/307 (7.8%)	9/66 (13.6%)	1/5 (20.0%)	**§0.254**	8/89 (9.0%)	18/243 (7.4%)	12/112 (10.7%)	4/23 (17.4%)	**0.371**
Spontaneous vaginal delivery	484/591 (81.9%)	92/106 (86.8%)	293/352 (83.2%)	52/71 (73.2%)	4/6 (66.7%)	**§0.067**	92/106 (86.8%)	237/285 (83.2%)	91/115 (79.1%)	21/29 (72.4%)	**0.224**
Caesarean section	86/598 (14.4%)	11/106 (10.4%)	47/355 (13.2%)	15/73 (20.5%)	2/6 (33.3%)	**0.121**	11/106 (10.4%)	37/288 (12.8%)	19/117 (16.2%)	8/29 (27.6%)	**0.092**
LBW <2.5kg	19/577 (3.3%)	4/107 (3.7%)	7/347 (2.0%)	4/72 (5.6%)	0/6 (0%)	**§0.281**	4/107 (3.7%)	7/281 (2.5%)	3/116 (2.6%)	1/28 (3.6%)	**§0.732**
Macrosomia >4.0kg	10/577 (1.7%)	1/107 (0.9%)	5/347 (1.4%)	2/72 (2.8%)	2/6 (33.3%)	**§0.006** **	1/107 (0.9%)	3/281 (1.1%)	3/116 (2.6%)	3/28 (10.7%)	**§0.018***
SGA	26/204 (12.7%)	5/42 (11.9%)	15/113 (13.3%)	3/29 (10.3%)	0/3 (0%)	**§1.000**	5/42 (11.9%)	15/99 (15.2%)	1/32 (3.1%)	2/14 (14.3%)	**§0.318**
LGA	8/204 (3.9%)	1/42 (2.4%)	3/113 (2.7%)	4/29 (13.8%)	0/3 (0%)	**§0.081**	1/42 (2.4%)	2/99 (2.0%)	3/32 (9.4%)	2/14 (14.3%)	**§0.050**
Born < 37 weeks	40/217 (18.4%)	7/43 (16.3%)	26/118 (22.0%)	2/29 (6.9%)	0/3 (0%)	**§0.263**	7/43 (16.3%)	23/103 (22.3%)	4/33 (12.1%)	1/14 (7.1%)	**0.353**
Born > 42 weeks	27/217 (12.4 %)	4/43 (9.3%)	16/118 (13.6%)	3/29 (10.3%)	0/3 (0%)	**§0.872**	4/43 (9.3%)	15/103 (14.6%)	3/33 (9.1%)	1/14 (7.1%)	**§0.790**
Postpartum haemorrhage	33/542 (6.1%)	5/94 (5.3%)	20/327 (6.1%)	7/69 (10.1%)	1/6 (16.7%)	**§0.284**	5/94 (5.3%)	14/262 (5.3%)	12/113 (10.6%)	2/27 (7.4%)	**0.275**
Sutures excluding Caesarean section	240/484 (49.6%)	53/91 (58.2%)	143/294 (48.6%)	24/56 (42.9%)	3/4 (75.0%)	**§0.187**	53/91 (58.2%)	125/239 (52.3%)	37/96 (38.5%)	8/19 (42.1%)	**0.037***
Initial feed at breast	541/581 (93.1%)	102/106 (96.2%)	331/348 (95.1%)	59/73 (80.8%)	3/5 (60.0%)	**§0.000*** **	102/106 (96.2%)	268/281 (95.4%)	102/117 (87.2%)	23/28 (82.1%)	**0.002** **
Breastfeeding at discharge	441/564 (78.2%)	82/101 (81.2%)	274/342 (80.1%)	47/71 (66.2%)	3/5 (60.0%)	**§0.038***	82/101 (81.2%)	221/275 (80.4%)	84/115 (73.0%)	19/28 (67.9%)	**0.184**
Back pain	21/539 (3.9%)	4/96 (4.2%)	12/329 (3.6%)	3/67 (4.5%)	0/5 (0%)	**§0.856**	4/96 (4.2%)	10/265 (3.8%)	5/111 (4.5%)	0/25 (0%)	**§0.906**
Baby admitted to intensive care unit	9/522 (1.7%)	0/92 (0%)	4/320 (1.3%)	2/64 (3.1%)	1/5 (20.0%)	**§0.030***	0/92 (0%)	4/257 (1.6%)	1/109 (0.9%)	2/23 (8.7%)	**§0.065**

BMI, Body mass index
GWG, gestational weight gain
SD, standard deviation
n, number
AN, antenatal
LBW, low birth weight
SGA, small for gestational age
LGA, large for gestational age
ANOVA used for continuous data
Chi square used for categorical data
*= p<0.05, ** = p<0.01, *** = p<0.001
‡ Kruskal - Wallis Test for continuous variables due to non-normality of distribution within each category
§ Exact test used where Chi square assumptions violated (expected count <5 in >20% of cells)
