# Supplementary material for: Pre-pregnancy body mass index and gestational weight gain and their effects on pregnancy and birth outcomes: a cohort study in West Sumatra, Indonesia
Source: BMC Womens Health. 2017 Nov 9;17:102. doi: 10.1186/s12905-017-0455-2 (PMC5679340; doi:10.1186/s12905-017-0455-2)
Supplement: Supplementary file 2 — Pregnancy and birth outcomes in relation to various gestational weight gains according to IOM recommendations for all BMI groups combined based on international and Asian classifications. This gives a table of pregnancy and birth outcome proportions according to IOM weight gain category, when applied to each BMI classification system. (PDF 38 kb) [file 12905_2017_455_MOESM2_ESM.pdf]

Pregnancy and birth outcomes in relation to various gestational weight gains according to IOM recommendations for all BMI groups combined based on international and Asian classifications

	IOM weight gain recommendations applied to international BMI classification				IOM weight gain recommendations applied to Asian BMI classification			
	**Inadequate**	**Recommended**	**Excessive**	**Overall p value**	**Inadequate**	**Recommended**	**Excessive**	**Overall p value**
Mean birthweight (g)<br>SD<br>N	3110<br>379<br>285	3208<br>427<br>169	3338<br>388<br>60	‡ **0.000*** **	3087<br>370<br>259	3226<br>427<br>177	3309<br>396<br>78	‡ **0.000*** **
Mean number of AN visits<br>SD<br>N	10.0<br>3.8<br>273	10.2<br>3.4<br>163	10.9<br>3.2<br>58	‡ **0.073**	9.8<br>3.8<br>248	10.3<br>3.5<br>171	10.9<br>3.3<br>75	‡ **0.015***
Mean gestation at delivery<br>SD<br>N	38.9<br>2.6<br>107	40.1<br>2.2<br>57	39.7<br>2.5<br>18	‡ **0.053**	38.9<br>2.7<br>101	40.0<br>2.3<br>56	39.7<br>2.3<br>25	‡ **0.088**
Haemoglobin <11.0g/dl in trimester 2	103/221 (46.6%)	56/137 (40.9%)	21/49 (42.9%)	**0.558**	96/205 (46.8%)	58/139 (41.7%)	26/63 (41.3%)	**0.566**
Haemoglobin <11.0g/dl in trimester 3	85/228 (37.3%)	39/132 (29.5%)	16/48 (33.3%)	**0.326**	79/208 (38.0%)	41/139 (29.5%)	20/61 (32.8%)	**0.255**
Induction	19/250 (7.6%)	16/151 (10.6%)	7/51 (13.7%)	**0.310**	17/224 (7.6%)	15/162 (9.3%)	10/66 (15.2%)	**0.177**
Spontaneous vaginal delivery	241/289 (83.4%)	134/166 (80.7%)	49/61 (80.3%)	**0.714**	218/260 (83.8%)	144/178 (80.9%)	62/78 (79.5%)	**0.583**
Caesarean section	36/293 (12.3%)	29/167 (17.4%)	8/61 (13.1%)	**0.313**	31/264 (11.7%)	30/179 (16.8%)	12/78 (15.4%)	**0.305**
LBW <2.5kg	9/285 (3.2%)	6/169 (3.6%)	0/60 (0%)	**§0.417**	9/259 (3.5%)	5/177 (2.8%)	1/78 (1.3%)	**0.599**
Macrosomia >4.0kg	2/285 (0.7%)	6/169 (3.6%)	2/60 (3.3%)	**§0.058**	1/259 (0.4%)	6/177 (3.4%)	3/78 (3.8%)	**§0.020***
SGA	18/102 (17.6%)	4/56 (7.1%)	1/18 (5.6%)	**0.105**	18/97 (18.6%)	3/54 (5.6%)	2/25 (8.0%)	**0.054**
LGA	2/102 (2.0%)	3/56 (5.4%)	2/18 (11.1%)	**§0.122**	2/97 (2.1%)	1/54 (1.9%)	4/25 (16.0%)	**§0.014***
Born < 37 weeks	25/107 (23.4%)	5/57 (8.8%)	3/18 (16.7%)	**0.068**	24/101 (23.8%)	5/56 (8.9%)	4/25 (16.0%)	**0.066**
Born ≥ 42 weeks	8/107 (7.5%)	12/57 (21.1%)	2/18 (111%)	**0.039***	8/101 (7.9%)	11/56 (19.6%)	3/25 (12.0%)	**0.097**
Postpartum Haemorrhage	15/263 (5.7%)	9/157 (5.7%)	7/58 (12.1%)	**0.183**	13/236 (5.5%)	9/168 (5.4%)	9/74 (12.2%)	**0.097**
Sutures excluding CS	108/244 (44.3%)	77/135 (57.0%)	26/49 (53.1%)	**0.050**	99/221 (44.8%)	84/146 (57.5%)	28/61 (45.9%)	**0.049***
Initial feed at breast	269/284 (94.7%)	152/168 (90.5%)	55/61 (90.2%)	**0.169**	242/256 (94.5%)	163/178 (91.6%)	71/79 (89.9%)	**0.278**
Breastfeeding at discharge	210//275 (76.4%)	131/166 (78.9%)	46/59 (78.0%)	**0.820**	187/247 (75.7%)	139/176 (79.0%)	61/77 (79.2%)	**0.670**
Back pain	1/261 (0.4%)	12/161 (7.5%)	6/58 (10.3%)	**0.000*** **	1/235 (0.4%)	10/171 (5.8%)	8/74 (10.8%)	**0.000*** **
Baby admitted to intensive care unit	2/252 (0.8%)	3/157 (1.9%)	2/55 (3.6%)	**§0.178**	2/226 (0.9%)	2/167 (1.2%)	3/71 (4.2%)	**§0.161**

IOM, institute of medicine
BMI, Body mass index
SD, standard deviation
n, number
AN, antenatal
LBW, low birth weight
SGA, small for gestational age
LGA, large for gestational age
*= p<0.05, ** = p<0.01, *** = p<0.001
Chi-square test used for categorical data
‡ Kruskal - Wallis Test for continuous variables due to non-normality of distribution within each category
§ Exact test used where Chi-square test assumptions violated (expected count <5 in >20% of cells)
